# Lipocalin-type prostaglandin D synthase regulates light-induced phase advance of the central circadian rhythm in mice

**DOI:** 10.1038/s42003-020-01281-w

**Published:** 2020-10-08

**Authors:** Chihiro Kawaguchi, Norihito Shintani, Atsuko Hayata-Takano, Michiyoshi Hatanaka, Ai Kuromi, Reiko Nakamura, Yui Yamano, Yusuke Shintani, Katsuya Nagai, Soken Tsuchiya, Yukihiko Sugimoto, Atsushi Ichikawa, Yasushi Okuno, Yoshihiro Urade, Hiroyuki Hirai, Kin-ya Nagata, Masataka Nakamura, Shuh Narumiya, Takanobu Nakazawa, Atsushi Kasai, Yukio Ago, Kazuhiro Takuma, Akemichi Baba, Hitoshi Hashimoto

**Affiliations:** 1 Laboratory of Molecular Neuropharmacology, Graduate School of Pharmaceutical Sciences, Osaka University, 1-6 Yamadaoka, Suita, Osaka 565-0871 Japan; 2 Molecular Research Center for Children’s Mental Development, United Graduate School of Child Development, Osaka University, Kanazawa University, Hamamatsu University School of Medicine, Chiba University and University of Fukui, 2-2 Yamadaoka, Suita, Osaka 565-0871 Japan; 3 Institute for Protein Research, Osaka University, 3-2 Yamadaoka, Suita, Osaka Japan; 4 Department of Physiological Chemistry, Graduate School of Pharmaceutical Sciences, Kyoto University, 46-29 Yoshida Shimoadachi-cho, Sakyo-ku, Kyoto 606-8501 Japan; 5 Department of Pharmaceutical Biochemistry, Kumamoto University Graduate School of Pharmaceutical Sciences, Oe-Honmachi, Kumamoto 862-0973 Japan; 6 Institute for Biosciences, Mukogawa Women’s University, 11-68 Koshien-Kyubancho, Nishinomiya-shi, Hyogo 663-8179 Japan; 7 Department of Biomedical Data Intelligence, Graduate School of Medicine, Kyoto University, Shogoin-Kawaharacho, Sakyo-ku, Kyoto, 606-8507 Japan; 8 Isotope Science Center, The University of Tokyo, 2-11-16 Yayoi, Bunkyo-ku, Tokyo, 113-0032 Japan; 9 Daiichi University of Pharmacy, 22-1 Tamagawa-machi, Minami-ku, Fukuoka, 815-8511 Japan; 10 Department of Advanced Medicine and Development, Bio Medical Laboratories Inc., 1361-1 Matoba, Kawagoe, Saitama, 350-1101 Japan; 11 Human Gene Sciences Center, Tokyo Medical and Dental University, 1-5-45 Yushima, Bunkyo-ku, Tokyo, 113-8510 Japan; 12 Department of Drug Discovery Medicine, Medical Innovation Center, Kyoto University Graduate School of Medicine, 53 Shogoin-Kawara-cho, Sakyo-ku, Kyoto, 606-8507 Japan; 13 Laboratory of Pharmacology, Graduate School of Dentistry, Osaka University, 1-8 Yamadaoka, Suita, Osaka 565-0871 Japan; 14 Department of Bioscience, Tokyo University of Agriculture, 1-1-1 Sakuragaoka, Setagaya-ku, Tokyo, 156-8502 Japan; 15 Laboratory of Biopharmaceutics, Graduate School of Pharmaceutical Sciences, Osaka University, 1-8 Yamadaoka, Suita, Osaka 565-0871 Japan; 16 Department of Cellular and Molecular Pharmacology, Graduate School of Biomedical and Health Sciences, Hiroshima University, 1-2-3 Kasumi, Minami-ku, Hiroshima, 734-8553 Japan; 17 Faculty of Pharmaceutical Sciences, Hyogo University of Health Science, 1-3-6 Minatojima, Chuo-ku, Kobe, Hyogo 650-8530 Japan; 18 Division of Bioscience, Institute for Datability Science, Osaka University, 1-1 Yamadaoka, Suita, Osaka 565-0871 Japan; 19 Transdimensional Life Imaging Division, Institute for Open and Transdisciplinary Research Initiatives, Osaka University, 2-1 Yamadaoka, Suita, Osaka 565-0871 Japan; 20 Department of Molecular Pharmaceutical Science, Graduate School of Medicine, Osaka University, 2-2 Yamadaoka, Suita, Osaka 565-0871 Japan

**Keywords:** Neurotrophic factors, Neuroscience, Circadian rhythms and sleep, Circadian regulation, Sleep

## Abstract

We previously showed that mice lacking pituitary adenylate cyclase-activating polypeptide (PACAP) exhibit attenuated light-induced phase shift. To explore the underlying mechanisms, we performed gene expression analysis of laser capture microdissected suprachiasmatic nuclei (SCNs) and found that lipocalin-type prostaglandin (PG) D synthase (L-PGDS) is involved in the impaired response to light stimulation in the late subjective night in PACAP-deficient mice. L-PGDS-deficient mice also showed impaired light-induced phase advance, but normal phase delay and nonvisual light responses. Then, we examined the receptors involved in the response and observed that mice deficient for type 2 PGD_2_ receptor DP2/CRTH2 (chemoattractant receptor homologous molecule expressed on Th2 cells) show impaired light-induced phase advance. Concordant results were observed using the selective DP2/CRTH2 antagonist CAY10471. These results indicate that L-PGDS is involved in a mechanism of light-induced phase advance via DP2/CRTH2 signaling.

## Abstract (web summary)

Kawaguchi et al. show that mice deficient in lipocalin-type prostaglandin (PG) D synthase (L-PGDS) exhibit impaired light-induced phase advance, but normal phase delay and nonvisual light responses. This study suggests the role of L-PGDS for the light-induced phase advance possibly via a chemoattractant receptor DP2/CRTH2.

## Introduction

The mammalian circadian clock system comprises the endogenous master pacemaker located within the suprachiasmatic nucleus (SCN) in the hypothalamus and coordinates physiology and behavior in relation to the 24 h day/night cycle. At the molecular level, it has been established that interlocked feedback loops of transcriptional activation by the CLOCK/BMAL1 complex and repression by the PER/CRY complex integrate with diverse environmental and metabolic stimuli to generate internal 24 h timing^1–4^. The primary input stimulus of the mammalian circadian clock is light, but feeding, temperature, and social cues can also be entrainment factors^3^. Clock disruption is primarily associated with sleep disorders but is also implicated in various diseases, including mental, metabolic, cardiovascular, and inflammatory disorders. Studies on clock dysfunctions therefore aim to unravel mechanisms and reveal potential novel therapeutic targets for these disorders^5,6^.

Previously, we demonstrated that mice lacking pituitary adenylate cyclase-activating polypeptide (PACAP)^7,8^ (*PACAP*^*−/−*^ mice) show an impairment in the light-dependent synchronization of diurnal rhythms (photic entrainment)^9,10^. A subset of retinal ganglion cells that express the circadian photopigment melanopsin also coexpress PACAP and glutamate, both of which have phase-shifting effects on the endogenous rhythm via regulation of clock gene expression in the SCN^11,12^. Thus, *PACAP*^*−/−*^ mice are considered to be a unique animal model to further investigate mechanisms of the generation and entrainment of the circadian rhythm. However, fundamental insight into the molecular basis of photic entrainment by PACAP signaling remains elusive.

Here, we performed gene chip analysis of laser capture-microdissected SCNs from *PACAP*^*−/−*^ and wild-type mice that had been kept in constant dark (DD) or exposed to light stimulation in the late subjective night to identify which genes were differentially expressed and categorize them by expression patterns. The analysis revealed that an increase in lipocalin-type prostaglandin (PG) D synthase (L-PGDS)^13,14^ expression in response to light stimulation in the late subjective night was not observed in *PACAP*^*−*^^*/−*^ mice. In the brain, L-PGDS and its enzymatic product PGD_2_ have been well established to play a crucial role in the regulation of physiological sleep^15,16^. However, the function of L-PGDS, as well as two subtypes of the PGD_2_ receptor, namely, DP1 and DP2/CRTH2 (chemoattractant receptor-homologous molecule expressed on Th2 cells; also known as GPR44), in the circadian clock remains unknown. In the present study, we therefore examined circadian rhythms and light-induced phase shifts in mice lacking L-PGDS (*L-PGDS*^*−/−*^), DP1 (*DP1*^*−*^^*/−*^), or DP2/CRTH2 (*CRTH2*^*−/−*^). L-PGDS and DP2/CRTH2-deficient mice showed impaired phase advance under low intensity light. These results indicate that L-PGDS is involved in a mechanism of light-induced phase advance via DP2/CRTH2 signaling.

## Results

### Identification of candidate genes involved in phase advance

We have previously observed that *PACAP*^*−*^^*/−*^ mice show severe dysfunctions of photic entrainment^10^, particularly in the abolishment of phase advance with low intensity (20 lx) light stimulation^9^. Therefore, in this study, we addressed downstream pathways that underpin circadian entrainment and performed gene chip analysis of laser capture-microdissected SCNs (Supplementary Fig. 1a) from four mouse groups: *PACAP*^*−*^^*/−*^ and wild-type mice, which were either illuminated with light in the late subjective night, circadian time (CT) 21, or kept without light (SCNs from 3 mice per group).

**Fig. 1:**
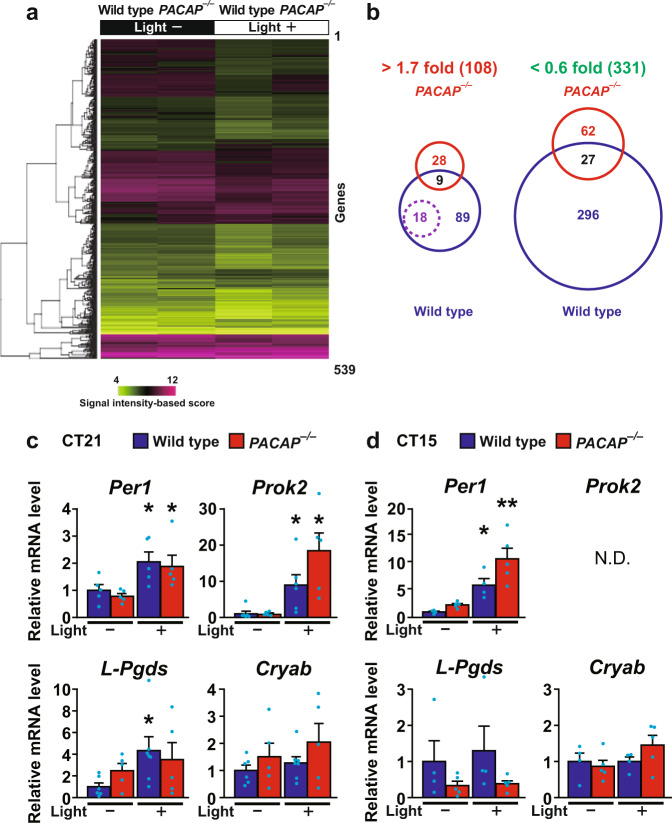
Gene chip analysis of laser capture-microdissected SCNs in *PACAP*^*−*^^*/−*^ and wild-type mice illuminated or not illuminated with light in the late subjective night (CT 21). **a** mRNA expression levels of the 539 differentially expressed genes as measured by hybridization signal intensity. Clustering dendrograms show the relative expression values according to the scale shown on the bottom left side (magenta, high expression level; light green, low expression level). **b** Venn diagram illustrating pairwise overlap of the genes. Data in each genotype comparison with light stimulation represent upregulated (>1.7-fold change) or downregulated (>0.6-fold change) genes. The 18 genes annotated to the term “extracellular region” are indicated by a purple dotted circle. These genes were upregulated by light stimulation in wild-type mice and are listed in Table 1. Real-time quantitative PCR analysis for *L-Pgds* and *Cryab* in the SCN upon light stimulation at CT 21 (**c**) and CT 15 (**d**). The *Per1* and *Prok2* genes were used as positive controls. ND indicates not detected. The values are shown as the mean ± SEM (*n* = 4–7 per group). Statistically significant differences were assessed using two-way ANOVA followed by Tukey–Kramer tests. *p < 0.05; **p < 0.01, vs kept without light stimulation.

Among the ~22,000 genes represented on the oligonucleotide array, 539 genes with hybridization signal ratios with a more than 1.7-fold change compared to the values for wild-type mice kept without light were selected and regarded as differentially expressed genes (Fig. 1a). Of these 593 genes, we specifically analyzed genes that were upregulated (>1.7-fold change) or downregulated (>0.6-fold change) by light stimulation in *PACAP*^*−*^^*/−*^ and wild-type mice. Of these genes, 108 (wild type only: 89 genes, *PACAP*^*−/−*^ only: 28 genes, both: 9 genes) were upregulated, while 331 genes (wild type only: 296 genes, *PACAP*^*−*^^*/−*^ only: 62 genes, both: 27 genes) were downregulated, under light stimulation (Fig. 1b).

Using the *k*-means clustering algorithm, the 539 genes were classified into 6 clusters on the basis of the similarity of the expression profiles (Supplementary Fig. 1b). Genes in cluster 1 were slightly downregulated in *PACAP*^*−/−*^ mice kept without light and upregulated by light stimulation compared with the levels in wild-type mice kept without light. Genes in cluster 2 show increased expression in *PACAP*^*−/−*^ mice kept without light and in both wild-type and *PACAP*^*−/−*^ mice under light stimulation compared with the expression in wild-type mice kept without light. Genes in clusters 3 and 4 show decreased expression in both wild-type and *PACAP*^*−/−*^ mice by light stimulation compared with the expression in wild-type mice kept without light. The difference between these clusters is the differential expression levels in *PACAP*^*−/−*^ mice under light stimulation. Genes in cluster 3 show decreased expression levels in both wild-type and *PACAP*^*−/−*^ mice under light stimulation. Genes in cluster 4 show decreased expression levels in wild-type mice compared with those in *PACAP*^*−/−*^ mice under light stimulation. Genes in cluster 5 were slightly upregulated in *PACAP*^*−/−*^ mice kept without light and downregulated by light stimulation in both wild-type and *PACAP*^*−/−*^ mice compared with wild-type mice kept without light. Cluster 6 includes genes that do not belong to cluster 1–5. To gain further insight into the clusters, we analyzed the informative gene ontology terms for each cluster (Supplementary Table 1). We found that genes in these clusters were categorized into partially overlapping but different gene ontology classifications.

In addition, gene ontology enrichment analysis for functional classification of light-responsive genes differentially expressed in wild-type mice showed that genes annotated to the term “binding” were the vast majority (48%), including immediate early response genes such as *Per1* and *fos* (Supplementary Fig. 1c). Gene ontology enrichment analysis for cellular components of light-responsive genes in wild-type and *PACAP*^*−/−*^ mice showed that the ratio of the number of light-responsive genes annotated to the term “extracellular region” to the total number of light-responsive genes was the most different between *PACAP*^*−/−*^ and wild-type mice (Supplementary Fig. 1d). Interestingly, most of the “extracellular region” genes were the 18 genes (indicated by a purple dotted circle in Fig. 1b and listed in Table 1) which were upregulated by light stimulation in wild-type mice but not in *PACAP*^*−/−*^ mice. Of these 18 genes, *L-Pgds* (*Ptgds*) showed the second largest change in expression in response to light stimulation in wild-type mice (Table 1).

**Table 1:** Genes showing upregulation in response to light at CT 21 in wild-type mice but not in *PACAP*^*−/−*^ mice.

Gene symbol	Gene name	GenBank accession #	WT light (+) vs WT light (−) log2 (fold)	KO light (−) vs WT light (−) log2 (fold)	KO light (+) vs WT light (−) log2 (fold)
*Purb*	Purine rich element binding protein B	AF017630	1.51	0.79	1.32
*Ptgds*	Prostaglandin D2 synthase (brain), Lipocalin type	AB006361	1.34	0.93	0.61
*Cryab*	Crystallin, alpha B	BC094033	1.21	0.98	0.93
*Hba-a 1*	Hemoglobin alpha, adult chain 1	L75940	1.15	1.21	1.24
*Mal*	Myelin and lymphocyte protein, T-cell differentiation protein	AK019046	1.09	0.99	1.13
*Ppp1r14a*	Protein phosphatase 1, regulatory (inhibitor) subunit 14A	AF352573	1.04	1.17	0.92
*Phgdh*	3-phosphoglycerate dehydrohenase	AA561726	1.04	0.87	0.75
*1110002E23 Rik*	RIKEN cDNA 1110002E23 gene	BB453951	1.01	0.87	1.16
*Ppp1r11*	Protein phosphatase 1, regulatory (inhibitor) subunit 11	BC027737	0.98	1.01	0.68
*Copg2as2*	Coatomer protein complex, subunit gamma 2, antisense 2	U20265	0.98	0.95	1.32
*Erdr1*	Erythroid differentiation regulator 1	AJ007909	0.95	0.8	1.22
*Rohn*	Ras homolog gene family, member N	AK075970	0.9	0.8	0.64
*1110008P14Rik*	RIKEN cDNA 1110008P14 gene	C79326	0.89	0.79	0.38
*Igf2*	Insulin-like growth factor 2	M14951	0.87	0.82	1.02
*Mobp*	Myelin-associated oligodendrocytic basic protein	AK013799	0.84	1.16	1.09
*Gfap*	Glial fibrillary acidic protein	AF332061	0.8	1.32	1.5
*Fmod*	Fibromodulin	BB483571	0.79	1.07	1.14
*Gsn*	Gelsolin	AV025559	0.78	0.82	0.75

Expression levels are indicated as the logarithm of fold change. Representative genes with the largest changes are shown.

*WT* wild-type mice, *KO* *PACAP*^−/−^ mice, *Light (+)* light stimulation, *Light (−)* kept without light.

Due to the considerable biological interest in L-PGDS, we performed real-time quantitative PCR using amplified RNA from laser-captured microdissected SCNs. We observed that *L-Pgds* was the only gene whose expression was increased by light at CT 21 in wild-type mice but not in *PACAP*^*−/−*^ mice [two-way analysis of variance (ANOVA), gene effect: *F*_(1,19)_ = 0.089, *p* = 0.77; light effect: *F*_(1,19)_ = 3.86, *p* = 0.06; interaction: *F*_(1,19)_ = 1.083, *p* = 0.31]. In addition, light-induced upregulation of *L-Pgds* expression was not observed in either wild-type or *PACAP*^*−/−*^ mice under light stimulation at CT 15 [two-way ANOVA, gene effect: *F*_(1,14)_ = 3.83, *p* = 0.07; light effect: *F*_(1,14)_ = 0.19, *p* = 0.67; interaction: *F*_(1,14)_ = 0.094, *p* = 0.76] (Fig. 1c, d). On the other hand, the changes in the expression of *Cryab* were not significant in wild-type and *PACAP*^*−/−*^ mice under light stimulation at CT 15 and CT 21 or without stimulation [two-way ANOVA, CT 15, gene effect: *F*_(1,14)_ = 0.52, *p* = 0.48; light effect: *F*_(1,14)_ = 1.83, *p* = 0.198; interaction: *F*_(1,14)_ = 1.83, *p* = 0.198; CT 21, gene effect: *F*_(1,19)_ = 2.38, *p* = 0.14; light effect: *F*_(1,19)_ = 0.98, *p* = 0.33; interaction: *F*_(1,19)_ = 0.10, *p* = 0.75]. The discrepancy between the microarray and RT-PCR results may be explained by the extremely low expression level of *Cryab* in the SCN. As expected, the expression of *Per1* and *Prok2*, which were positive targets, was significantly increased in wild-type and *PACAP*^*−/−*^ mice under light stimulation either at CT 15 [two-way ANOVA, *Per1*, gene effect: *F*_(1,14)_ = 7.18, *p* = 0.018; light effect: *F*_(1,14)_ = 24.96, *p* = 0.0002; interaction: *F*_(1,14)_ = 1.24, *p* = 0.28; *Prok2*, not detected] or at CT 21 [two-way ANOVA, *Per1*, gene effect: *F*_(1,18)_ = 0.44, *p* = 0.52; light effect: *F*_(1,18)_ = 12.89, *p* = 0.002; interaction: *F*_(1,18)_ = 0.014, *p* = 0.91; *Prok2*, gene effect: *F*_(1,18)_ = 2.43, *p* = 0.14; light effect: *F*_(1,18)_ = 18.35, *p* = 0.0004; interaction: *F*_(1,18)_ = 2.63, *p* = 0.12].

### L-PGDS localization in the SCN

**Fig. 2:**
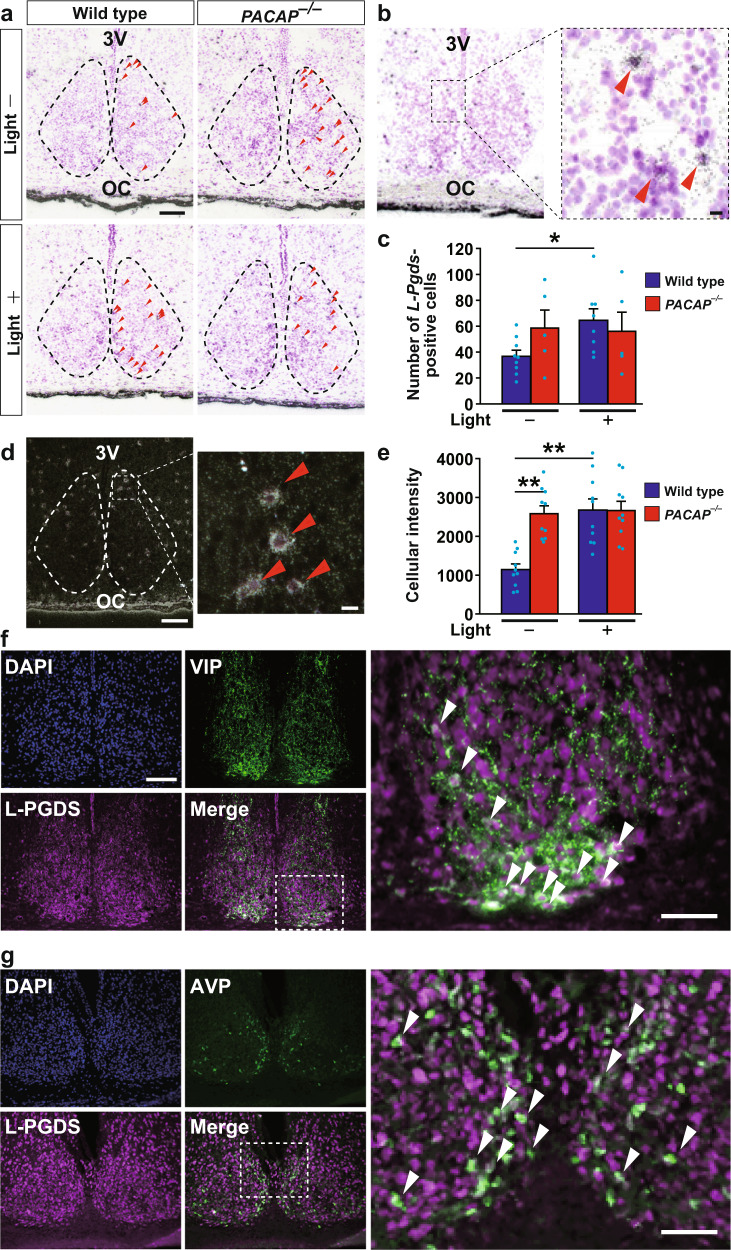
*L-Pgds (Ptgds)* expression in the SCN in the late subjective night. **a** In situ hybridization with a [^35^S]CTP-labeled antisense probe for *L-Pgds* in the SCN at CT 21. Representative bright-field photomicrographs are shown for *PACAP*^*−/−*^ and wild-type mice, which were either light stimulated (light+) at CT 21 or kept without light (light−). 3V third ventricle; OC optic chiasma. The dotted line indicates the borders of the SCN area that contain densely aggregated cresyl violet-stained nuclei. Red arrowheads in the right SCN region indicate *L-Pgds* signals that were merged with counterstained neurons. Bar, 100 μm. **b** Wild-type mice illuminated with light. Right panel, magnification of the area marked with a black box. Red arrowheads indicate *L-Pgds* signals that were merged with counterstained neurons. Bars, 10 μm. **c** The number of *L-Pgds*-positive cells quantified in the SCN. To quantitatively determine the *L-Pgds*-expressing neurons in the whole SCN, five coronal SCN sections every four sections per mouse were used for statistical analysis. The values are shown as the mean ± SEM (*n* = 5–9 per group). *p < 0.05. Statistically significant differences were assessed using two-way ANOVA followed by Tukey–Kramer tests. **d** Representative dark-field photomicrographs. Bar, 100 μm. Right panel, magnification of the area marked with a white box. Red arrowheads indicate *L-Pgds* signals on individual cell bodies that were merged with Nissl-stained neurons. Bar, 10 μm. **e** Intensity of in situ hybridization signals for *L-Pgds* in each cell in the SCN. The values are shown as the mean ± SEM (*n* = 10 per group). **p < 0.01. Statistically significant differences were assessed using two-way ANOVA followed by Tukey–Kramer tests. Double immunohistochemical staining for L-PGDS (magenta) and VIP (green, **f**) or AVP (green, **g**) in the SCN. Right panels, magnification of the areas marked with white boxes. White arrowheads, cells that were immunoreactive for L-PGDS and VIP or AVP. Bars, 100 μm.

Of the genes altered at CT 21, only *L-Pgds* showed differences in expression between wild-type and *PACAP*^*−/−*^ mice (Fig. 1c). Under DD conditions, the expression of *L-Pgds* showed a tendency to be high at CT 15 and low at CT 21 in wild-type mice, while the opposite trend was observed in *PACAP*^*−/−*^ mice (Supplementary Fig. 2a). The lack of induction of *L-Pgds* by light at CT 21 in *PACAP*^*−/−*^ mice was considered to result from the elevated basal expression of *L-Pgds* in the SCN in these mutant mice. L-PGDS is known to produce PGD_2_, which is the most abundant prostanoid in the brains^16^. In situ hybridization of *L-Pgds* signals was detected as intense clusters of black grains under bright-field illumination (Fig. 2a, b) as well as white grains under dark-field illumination (Fig. 2d). We counted the number of *L-Pgds* signals on individual cell bodies that were merged with Nissl-stained neurons in the SCN using ImageJ software (NIH) (Fig. 2b). Although the number of *L-Pgds*-expressing cells tended to increase upon illumination at CT 21 in wild-type mice, the results did not show statistically significant differences [two-way ANOVA, gene effect: *F*_(1,23)_ = 0.45, *p* = 0.51; light effect: *F*_(1,23)_ = 1.60, *p* = 0.22; interaction: *F*_(1,23)_ = 2.32, *p* = 0.14] (Fig. 2c). Then, we counted the cellular intensity of *L-Pgds* expression in the SCN. The cellular intensity of *L-Pgds* expression was significantly increased by light at CT 21 in wild-type mice [two-way ANOVA, gene effect: *F*_(1,36)_ = 10.11, *p* = 0.003; light effect: *F*_(1,36)_ = 12.97, *p* = 0.001; interaction: *F*_(1,36)_ = 10.47, *p* = 0.003] but not in *PACAP*^*−/−*^ mice (Fig. 2d, e). In contrast, there was no difference in the number of *L-Pgds*-positive neurons in the outside vicinity of the SCN with or without light stimulation between genotypes [*L-PGDS*^*−/−*^; light stimulant, 272 ± 35 cells; kept without light, 251 ± 24 cells; wild type; light stimulant, 308 ± 26 cells; kept without light, 251 ± 18 cells, not significant, two-way ANOVA] (Supplementary Fig. 2b). We also performed double immunostaining of L-PGDS with NeuN or Olig2 and observed that L-PGDS immunoreactivity was mainly localized in NeuN-positive neurons and partly in Olig2-positive oligodendrocytes (Supplementary Fig. 2c, d). In addition, double immunostaining of L-PGDS with vasoactive intestinal peptide (VIP) or vasopressin (AVP) (Fig. 2f, g) showed that VIP-immunoreactive neurons were localized exclusively in the ventrolateral core region in the SCN and their fibers were distributed throughout the SCN (Fig. 2f), while AVP-immunoreactive neurons were localized mostly in the dorsomedial shell part in the SCN (Fig. 2g). The roles of SCN core and shell are known to be functionally distinct^17^. L-PGDS-immunoreactivity was localized with both VIP and AVP throughout the SCN (Fig. 2d, e). The immunoreactivity of L-PGDS was localized in the perinucleus, such as the nuclear envelope, Golgi apparatus, and secretory vesicles. In contrast, the immunoreactivity of VIP or AVP was localized in the cytoplasm and fibers. Although the subcellular localization differed between L-PGDS and VIP or AVP, these signals partly merged and seemed to be in the same neurons. Therefore, we assume that L-PGDS and VIP or AVP were colocalized in the neurons (Fig. 2f, g).

### Impaired light-induced phase advance in *L-PGDS*^*−/−*^ mice

**Fig. 3:**
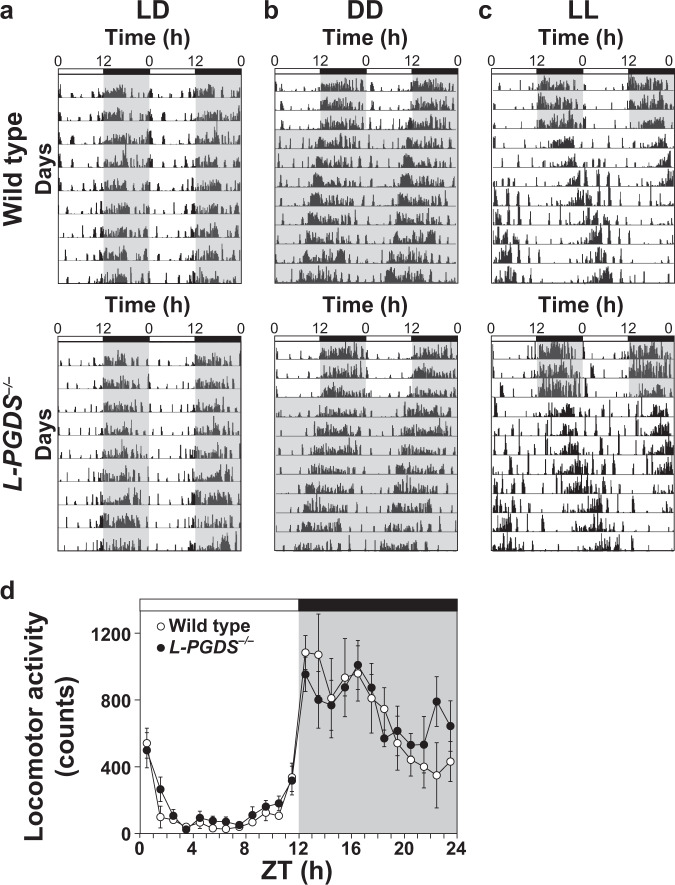
Circadian rhythms of locomotor activities in wild-type and *L-PGDS*^*−/−*^ mice under light-dark (LD), constant dark (DD) and constant light (LL) conditions. Representative double-plotted actograms of wild-type and *L-PGDS*^*−/−*^ mice kept in the LD cycle (light, 12 h; dark, 12 h) (**a**) or transferred from LD to DD (**b**) or LL (**c**). **d** Daily variations in locomotor activities of wild-type and *L-PGDS*^*−/−*^ mice under LD conditions. Intensity of illumination during the light phase, 100 lx. The values are expressed as the mean ± SEM (*n* = 5 per group). Statistically significant differences were assessed using two-way repeated measures ANOVA followed by Tukey–Kramer tests.

We subsequently examined circadian rhythms of locomotor activity in *L-PGDS*^*−/−*^ mice. *L-PGDS*^*−/−*^ mice were normally synchronized with a light cycle of 12 h of light and 12 h of dark (LD) and retained behavioral periodicity under DD conditions compared with wild-type mice (Fig. 3a, b, Table 2). However, under constant light (LL) condition, *L-PGDS*^*−/−*^ mice showed slightly but significantly increased duration of free-running period (Fig. 3c, Table 2). The pattern and magnitude of locomotor activity during an LD cycle was not different between *L-PGDS*^*−/−*^ and wild-type mice [two-way repeated measures ANOVA, gene effect, *F*_(1,8)_ = 0.26, *p* = 0.62; time effect: *F*_(23,184)_ = 20.59, *p* < 0.0001; interaction: *F*_(23,184)_ = 0.80, *p* = 0.73] (Fig. 3d).

**Table 2:** Parameters of circadian rhythm under the DD and LL conditions in *L-PGDS*^*−/−*^ mice.

	Wild type	*L-PGDS*^*−/−*^	*p* value
DD			
Tau (hour)	23.69 ± 0.55	23.64 ± 0.55	NS
Power	609.21 ± 16.38	602.60 ± 27.57	NS
Average activities	406.78 ± 18.23	489.13 ± 34.21	NS
*α* (hour)	10.13 ± 0.73	10.36 ± 0.62	NS
*α*/*ρ*	0.80 ± 0.12	0.81 ± 0.08	NS
LL			
Tau (hour)	25.23 ± 0.14	25.74 ± 0.14	<0.05
Power	522.23 ± 40.93	530.06 ± 30.76	NS
Average activities	220.65 ± 20.45	285.50 ± 39.50	NS
*α* (hour)	5.31 ± 0.81	5.28 ± 0.71	NS
*α*/*ρ*	0.27 ± 0.05	0.26 ± 0.04	NS

The free-running period (Tau), power, average activities, activity time (*α*) and *α*/*ρ* (activity-rest) ratio were examined. The values are expressed as the mean ± SEM (*n* = 8–10 per group).

*NS* not significant.

*p < 0.05. Statistically significant differences were assessed using one-way ANOVA followed by Tukey–Kramer tests.

**Fig. 4:**
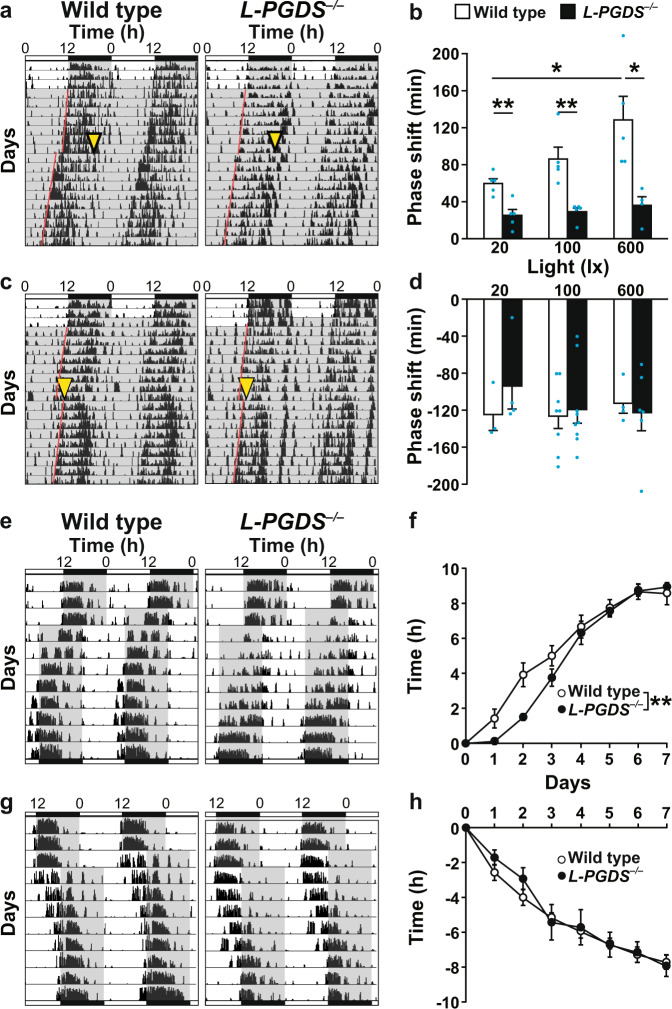
Impairment in light-induced phase advance in *L-PGDS*^*−/−*^ mice. Phase shift induced by light stimulation at CT 21 (**a**, **b**) and CT 15 (**c**, **d**) in *L-PGDS*^*−/−*^ and wild-type mice. **a**, **c** Representative double-plotted actograms. Yellow arrowheads indicate light stimulation (20 lx, 30 min). Paired red lines represent the onset of activity. Quantification of the phase shift induced by light stimulation. The values are expressed as the mean ± SEM (*n* = 4–5 (**b**), *n* = 3–9 (**d**) per group). *p < 0.05; **p < 0.01. Statistically significant differences were assessed using two-way ANOVA followed by Tukey–Kramer tests. Resynchronization of circadian rhythms to time shifts, 8 h advance (**e**, **f**) and 8 h delay (**g**, **h**). **e**, **g** Representative patterns of wheel running activity. **f**, **h** Quantification of the phase shift. The values are expressed as the mean ± SEM (*n* = 6–8 per group). **p < 0.01. Statistically significant differences were assessed using two-way repeated measures ANOVA.

In contrast, *L-PGDS*^*−/−*^ mice showed impaired photic entrainment function. A light pulse (20, 100, or 600 lx, 30 min) in the late subjective night (CT 21) induced phase advance of locomotor activity rhythms at significantly lower levels in *L-PGDS*^*−/−*^ mice than in wild-type mice [two-way ANOVA, gene effect: *F*_(1,23)_ = 32.65, *p* < 0.0001; light effect: *F*_(2,23)_ = 4.54, *p* = 0.022; interaction: *F*_(2,23)_ = 2.39, *p* = 0.11] (Fig. 4a, b and Supplementary Fig. 3), while a light pulse in the early subjective night (CT 15) induced phase delay in *L-PGDS*^*−/−*^ and wild-type mice at similar levels [two-way ANOVA, gene effect: *F*_(1,28)_ = 0.36, *p* = 0.55; light effect: *F*_(2,28)_ = 0.27, *p* = 0.77; interaction: *F*_(2,28)_ = 0.49, *p* = 0.62] (Fig. 4c, d).

Using a jet lag model in which mice were exposed to an 8 h time shift (advance or delay), we examined the synchronization of circadian rhythms in *L-PGDS*^*−/−*^ mice. When the light cycle was advanced by 8 h, *L-PGDS*^*−/−*^ mice showed significantly delayed synchronization compared to wild-type mice [two-way repeated measures ANOVA, gene effect: *F*_(1,12)_ = 2.70, *p* = 0.13; day effect: *F*_(7,84)_ = 175.84, *p* < 0.0001; interaction: *F*_(7,84)_ = 3.16, *p* = 0.005] (Fig. 4e, f). In contrast, both *L-PGDS*^*−/−*^ and wild-type mice showed similar synchronization when the light cycle was delayed by 8 h [two-way repeated measures ANOVA, gene effect: *F*_(1,12)_ = 0.11, *p* = 0.75; day effect: *F*_(7,84)_ = 121.54, *p* < 0.0001; interaction: *F*_(7,84)_ = 1.01, *p* = 0.43] (Fig. 4g, h).

### *L-PGDS*^*−/−*^ mice show normal light-induced c-Fos expression

Light-induced c-Fos-expression was examined in the SCN in *L-PGDS*^*−/−*^ mice (Supplementary Fig. 4). Light stimulation at CT 21 markedly increased the number of c-Fos-immunoreactive cells in the SCN in both *L-PGDS*^*−/−*^ and wild-type mice. There was no difference in the distribution and number of c-Fos-immunoreactive cells under light stimulation between genotypes [*L-PGDS*^*−/−*^, 411.9 ± 39.07 cells; wild type, 453.6 ± 32.2 cells; two-way ANOVA, not significant].

### *L-PGDS*^*−/−*^ mice show a normal nonvisual light responses

To examine the integrity of visual pathways, we examined the pupillary light reflex in *L-PGDS*^*−/−*^ mice. There were no differences in pupil sizes under scotopic conditions between genotypes (*L-PGDS*^*−/−*^, 1.87 ± 0.06 mm^2^; wild type, 1.99 ± 0.08 mm^2^; Student’s *t* test, not significant) (Supplementary Fig. 5a, b). Light-induced (100 and 600 lx) light reflexes were not significantly different in *L-PGDS*^*−/−*^ and wild-type mice (Supplementary Fig. 5c, d).

Negative masking responses to light (nocturnal animals are normally passive in a high-illumination milieu^18^) were normal in *L-PGDS*^*−/−*^ mice (Supplementary Fig. 5e–g). The amount of activity in mice exposed to a 2-h light pulse (100 and 400 lx) during the early night (Zeitgeber time 13–15) was similarly suppressed in *L-PGDS*^*−/−*^ and wild-type mice [two-way ANOVA, 100 lx, gene effect: *F*_(1,26)_ = 1.03, *p* = 0.32; light effect: *F*_(1,26)_ = 17.14, *p* = 0.003; interaction: *F*_(1,26)_ = 0.21, *p* = 0.65; 400 lx, gene effect: *F*_(1,23)_ = 2.48, *p* = 0.13; light effect: *F*_(1,23)_ = 121.41, *p* < 0.0001; interaction: *F*_(1,23)_ = 0.13, *p* = 0.72].

### DP2/CRTH2 mediates light-induced phase advance

**Fig. 5:**
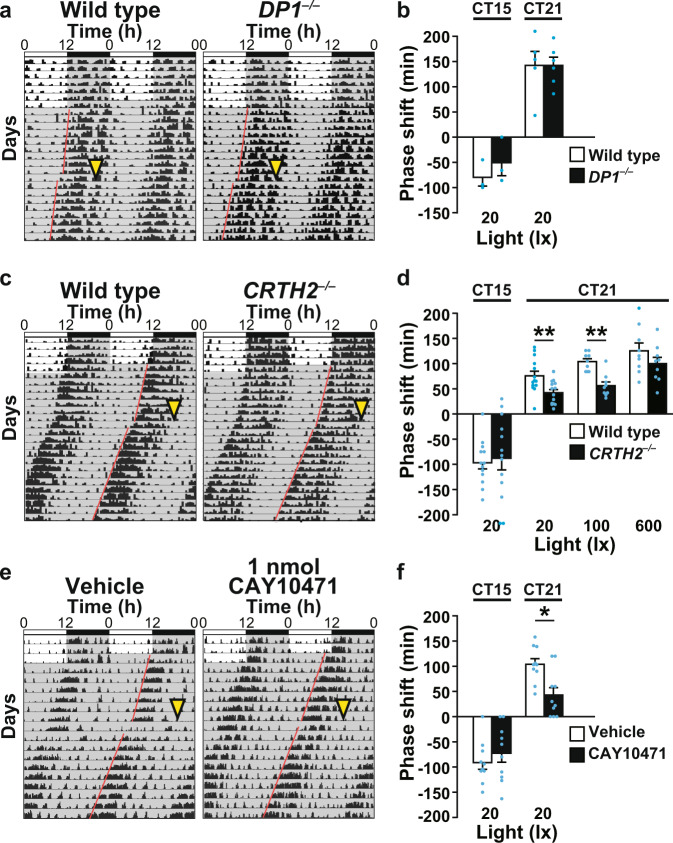
Impairment in light-induced phase advance in *CRTH2*^*−/−*^ mice and in mice administered the CRTH2 blocker CAY10471. Phase shift induced by light stimulation at CT 15 or CT 21 in *DP1*^*−/−*^ (**a**, **b**) and *CRTH2*^*−/−*^ (**c**, **d**) mice and wild-type mice of the respective genetic backgrounds. **a**, **c** Representative double-plotted actograms. Quantification of the phase shift induced by the indicated light stimulation. The values are expressed as the mean ± SEM (*n* = 3–6 (**b**), *n* = 9–14 (**d**) per group). **e**, **f** Phase shift induced by light stimulation at CT 15 or CT 21 in CD-1 wild-type mice administered the CRTH2 blocker CAY10471 or a vehicle (Riger’s solution) 30 min before light stimulation. **e** Representative double-plotted actograms. **f** Quantification of the phase shift. The values are expressed as the mean ± SEM (*n* = 10 per group). *p < 0.05; **p < 0.01. Statistically significant differences were assessed using two-way ANOVA followed by Tukey–Kramer tests. Yellow arrowheads indicate light stimulation (20 lx, 30 min). Paired red lines represent the onset of activity.

To determine which subtype of PGD_2_ receptors, i.e., DP1 or DP2/CRTH2, is responsible for light-induced phase advance, we examined circadian entrainment of locomotor activity in *DP1*^*−/−*^ and *CRTH2*^*−/−*^ mice. A light pulse (20 or 100 lx, 30 min) in the late subjective night (CT 21) induced phase advance of locomotor activity rhythms at significantly lower levels in *CRTH2*^*−/−*^ mice than in wild-type mice, although a stronger light pulse (600 lx, 30 min) at CT 21 induced similar levels of phase advance in *CRTH2*^*−/−*^ and wild-type mice [two-way ANOVA, gene effect: *F*_(1,58)_ = 1.03, *p* < 0.0001; light effect: *F*_(2,58)_ = 16.97, *p* < 0.0001; interaction: *F*_(2,58)_ = 0.60, *p* = 0.55] (Fig. 5c, f and Supplementary Fig. 3b). *CRTH2*^*−/−*^ mice showed normal levels of phase delay compared with wild-type mice (Fig. 5d). *DP1*^*−/−*^ mice showed normal levels of phase advance and delay even with exposure to very dim light (20 lx, 30 min) compared with wild-type mice (Fig. 5a, b).

We next examined the effect of intracerebroventricular administration of the DP2/CRTH2 antagonist CAY10471 or DP1 antagonist BW A868C on light-induced phase advance in wild-type mice in the CD-1 genetic background. CAY10471 significantly diminished light-induced (20 lx, 30 min) phase advance at CT 21 [two-way ANOVA, treat effect: *F*_(1,36)_ = 2.18, *p* = 0.15; light time effect: *F*_(1,36)_ = 116.20, *p* < 0.0001; interaction: *F*_(1,36)_ = 7.60, *p* = 0.009] (Fig. 5e, f). In contrast, BW A868C-treated mice showed normal levels of phase advance compared with vehicle-treated mice at each light pulse (20 or 600 lx) (Supplementary Fig. 6).

## Discussion

PACAP is cotransmitted with glutamate in melanopsin-containing retinal ganglion cells, which monosynaptically innervate the SCN, and mediates nonvisual photoreception-regulated light-induced circadian entrainment, negative masking of locomotor activity, and the pupillary light reflex^12,19,20^. Since *PACAP*^*−/−*^ mice show impaired light-induced circadian entrainment and negative masking and unusually early onset of activities during the light-to-dark transition period (an “early-bird” phenotype)^9,10^, in the present study, we conducted transcriptome analysis of laser capture-microdissected SCNs from *PACAP*^*−/−*^ and wild-type mice with or without light stimulation in the late subjective night. We identified that *L-Pgds* showed the second largest change between the illuminated mutant mice and basal wild-type mice (Table 1). It is intriguing that *L-Pgds* expression was increased by light specifically at CT 21. We found that the individual intensity of *L-Pgds* signals in each cell was significantly increased by light at CT 21 in the SCN in wild-type mice (Fig. 2e), suggesting that the increased *L-Pgds* levels (Fig. 1c) are likely attributable to the increased individual intensity of *L-Pgds* signals in each cell in the SCN. Fujimori et al. demonstrated that the promoter of *L-Pgds* has an E-box motif using a luciferase reporter assay^21^, suggesting that *L-Pgds* may show changes in circadian expression under light stimulation. The blunted induction of *L-Pgds* in *PACAP*^*−/−*^ mice by light at CT 21 might arise from abnormal basal expression of *L-Pgds* in the SCN in *PACAP*^*−/−*^ mice (Supplementary Fig. 2a). *Purb* showed the largest change between the illuminated mutant mice and basal wild-type mice (Table 1). Recent studies have shown that PURB is a single-stranded nucleic acid-binding protein that is involved in the regulation of DNA replication and transcription; however, little has been reported on its function in the central nervous system. Furthermore, *Purb*-deficient mice are not available. Therefore, although it is important to understand the role of PURB in the circadian system, we assume that it would take considerable effort to convincingly determine how PURB is involved in the phase advance of the circadian rhythm in response to the light pulse. The role of *Purb* and the other genes differentially expressed in the current study should be addressed in future research.

*L-PGDS*^*−/−*^ mice showed impaired phase advance under light at CT 21 but normal phase delay under light at CT 15. These results may implicate L-PGDS in phase advance-selective re-entrainment. *PACAP*^*−/−*^ mice have been shown to convey parametric light information depending on intensity and duration. PACAP and L-PGDS may thus be involved in a mechanism for directional asymmetry in circadian entrainment (where phase advance is impaired while phase delay is normal in *PACAP*^*−/−*^ and *L-PGDS*^−/−^ mice). Previous studies have found that *L-Pgds* gene expression was upregulated by protein kinase C through derepression of notch-HES signaling and augmentation of AP-2β in human TE671 (medulloblastoma of cerebellum) cells^22^. Moreover, a key prostanoid enzyme, cyclooxygenase-2, and PGE_2_ biosynthesis were induced by interleukin 1 beta (IL-1β) via protein kinase C activation and mitogen-activated protein kinases cascade in the glial cells^23^. PACAP has been shown to stimulate extracellular signal-regulated kinase 1/2 activity^24^. Future studies into the functional relationships between PACAP- and L-PGDS-mediated signaling pathways will reveal the precise role of PACAP in light-induced circadian entrainment.

In the present study, the light-induced c-Fos expression was normally observed in *L-PGDS*^*−/−*^ mice light stimulated at CT 21 (Supplementary Fig. 4), which is consistent with our previous result for *PACAP*^*−/−*^ mice^9^. These results suggest that c-Fos is not critically involved in the PACAP- and L-PGDS-mediated phase advance of the central circadian clock.

Considering our current results, DP2/CRTH2 is also not involved in a mechanism of light-induced phase advance under 600 lx light (Fig. 5c, d and Supplementary Fig. 3b). Thus, the impaired light-induced phase advance under 600 lx in *L-PGDS*^−/−^ mice may be explained by the PGD_2_ signaling-independent function of L-PGDS; however, the molecular mechanism underlying the function of L-PGDS is currently unclear. L-PGDS is reported to act as an extracellular transporter of various lipophilic small molecules as well as a PGD2-synthesizing enzyme^14^. L-PGDS binds to all-*trans*-retinoic acids that have been identified as potential circadian entrainment factors^25,26^. Moreover, Lee et al. reported a novel nonenzymatic function of L-PGDS, i.e., regulation of glial cell migration and morphology by binding to the MARCKS heat shock protein^27^. Alternatively, either one of the two PGD2 receptor subtypes may be sufficient to mediate PGD2 signaling for the light-induced phase advance under 600 lx light. Further analysis, e.g., using DP1 and DP2/CRTH2 double-deficient mice, is needed to precisely understand the function of L-PGDS and PGD2 receptors signaling pathway in the light-induced circadian entrainment.

It has been shown that PACAP, L-PGDS, DP1, and DP2/CRTH2 are expressed in SCN^11,12^; PACAP is expressed in neurons and astrocytes^28^; L-PGDS is expressed in leptomeningeal cells, neurons, and oligodendrocytes^29^; DP1 is expressed in neurons and microglia^30^; and DP2/CRTH2 is expressed mainly in astrocytes^29^. These data suggest that L-PGDS/PGD_2_ and downstream DP2/CRTH2 signaling may represent a mechanism that is linked to neural and astrocytic signaling cascade involved in light-induced phase advance of the central circadian clock. Future studies, e.g., cell type-specific single-cell transcriptome analysis, may reveal the precise mechanism.

In the brain, PGD_2_ acts as the most potent endogenous sleep-promoting substance reported thus far^16^ and is involved in PGE_2_-induced neuropathic pain^31^. DP1 receptor signaling is involved in these PGD_2_ actions. In a genetic demyelination model of *twitcher* mice, L-PGDS has been implicated in neural protection^32^, while hematopoietic PGD synthase (H-PGDS), which is responsible for the production of PGD_2_ in inflammatory responses, plays a role in the progression of neural inflammation^29^. The PGD_2_ concentration in rat cerebrospinal fluid shows circadian rhythmicity in parallel with the sleep–wake cycle^33^, and L-PGDS levels in human serum also show circadian rhythmicity, with a nocturnal increase^34^. In the cultured peripheral fibroblasts, 15-deoxy Δ[12, 14] PG J_2_ (15d-PGJ_2_) and PGJ_2_, a derivative of PGD_2_, were reported to reset the peripheral circadian clock^26,35^. However, the function of L-PGDS as well as the two PGD_2_ receptor subtypes DP1 and DP2/CRTH2 in the circadian clock remains unknown. To our knowledge, the present results provide the first evidence that L-PGDS-derived PGD_2_ and the downstream DP2/CRTH2 specifically mediate light-induced phase advance of the central circadian clock.

Although DP1 was originally identified as a homolog of other PG receptors^36^, DP2/CRTH2 is a member of the G protein-coupled leukocyte chemoattractant receptor family, which is selectively expressed in Th2 but not Th1 lineage cells, and is thereby named CRTH2 (chemoattractant receptor-homologous molecule expressed on Th2 cells)^37^. In contrast to the functions of DP1, the functions of DP2/CRTH2 in the brain are not well understood. Mohri et al. have reported that both DP1 and DP2/CRTH2 are expressed in cultured astrocytes, stimulation of which leads to enhanced GFAP production, suggesting that PGD_2_ plays an important role in microglia/astrocyte interactions^29^. Previously, we showed that 15d-PGJ_2_ enhances nerve growth factor-induced neurite outgrowth in vitro, through activation of DP2/CRTH2^38^. More recently, we showed that DP2/CRTH2 is critically involved in impairments of emotional aspects induced by lipopolysaccharide or tumor (colon 26) inoculation^39,40^ and cognitive dysfunction induced by the *N*-methyl-d-aspartate receptor antagonist MK-801^41^. These results suggest that DP2/CRTH2 antagonism has potential as a therapeutic target for behavioral symptoms. Because the circadian clock is closely associated with the sleep–wake cycle^1,2,5^, the present results may provide an additional mechanism for the somnogenic effect of PGD_2_ in that DP2/CRTH2-mediated PGD_2_ signaling regulates light-induced the central circadian entrainment.

Although it remains unclear how PGD_2_-DP2/CRTH2 signaling induces circadian entrainment, in NIH3T3 cells, 15d-PGJ_2_, a putative ligand of DP2/CRTH2, has been shown to trigger rhythmic endogenous clock gene expression and transiently upregulate *Cry1*, *Cry2*, and *Rorα* expressions^26^. Similar regulatory mechanism of clock gene expression may mediate circadian entrainment in which PGD2-DP2/CRTH2 signaling is involved.

In this study, we used three different mouse strains, *PACAP*^*−/−*^ in a CD-1 background, *L-PGDS*^*−/−*^ and *CRTH2*^*−/−*^ in a BALB/c background, and *DP1*^*−/−*^ in a C57BL/6 background. *PACAP*^*−/−*^ mice in the C57BL/6 background showed extremely high postnatal mortality^42^. Therefore, we backcrossed the null mutation onto the CD-1 mouse background. The difference between average time of phase shift at 20 lx in wild-type mice shown Figs. 4 and 5 might arise from the different between mouse strains (e.g., BALB/c, Fig. 4, vs C57BL/6, Fig. 5a, b). Schwartz reported that the two strains of BALB/c and C57BL/6 mice showed a large difference in the free-running period and phase advance during the late subjective night to early subjective day^43^. It is known that mouse strain differences may reflect a wide range of sensitivities to light, as eye color, thickness of outer nuclear layer in retina, melatonin deficiency and locomotor activity differ among mouse strains^44^. Although all mouse strains examined in this study showed phase advance during the late subjective night and we performed all experiments using wild-type and mutant mice with the same mouse background, the difference in the mouse backgrounds must be carefully taken into account to interpret the data obtained from different strains. Therefore, the present results observed in *DP1*^*−/−*^ mice must be carefully interpreted and scrutinized in future research.

In summary, we obtained the following main findings: (1) L-PGDS-deficient mice showed impaired phase advance of circadian rhythm locomotor activity but normal phase delay when assessed using the effect of light pulses in DD conditions or with a jet lag model in which mice were exposed to an 8 h time shift (advance or delay), while (2) the mutant’s other nonvisual light responses, including light-induced locomotor suppression and pupillary light reflex, were normal. (3) DP2/CRTH2-deficient mice showed impaired light-induced phase advance but normal phase delay. Finally, (4) the selective DP2/CRTH2 antagonist CAY10471 impaired light-induced phase advance. These results show that L-PGDS-derived PGD_2_ and downstream DP2/CRTH2 signaling constitute a novel signaling cascade specifically involved in light-induced phase advance of the central circadian clock. The results also provide insights into the roles of prostanoids in the regulation of brain functions.

## Methods

### Mice

All animal care and handling procedures were approved by the Animal Care and Use Committee of the Graduate School of Pharmaceutical Sciences, Osaka University. The generation of *PACAP*^*–/−*^^45^, *L-PGDS*^*−/−*^^31^, *DP1*^*−/−*^^46^, and *CRTH2*^*−/−*^^47^ mice via gene targeting has been previously reported; these mice were backcrossed for at least 10 generations onto the CD-1 (*PACAP*^*−/−*^), C57BL/6 (*DP1*^*−/−*^), or BALB/c (*L-PGDS*^*−/−*^ and *CRTH2*^*−/−*^) genetic background. Mice were kept under an LD cycle (light on from 8 a.m. to 8 p.m. unless otherwise specified) at a controlled room temperature. Pelleted food (CMF; Oriental Yeast, Osaka, Japan) and water were available ad libitum. Male knockout mice (*PACAP*^*−/−*^, *L-PGDS*^*−/−*^, *DP1*^*−/−*^, and *CRTH2*^*−/−*^) and wild-type mice of the respective genetic backgrounds were used for this study. All experiments were carried out on male mice at 6 to 12 weeks of age.

### Microarray analysis

Mice synchronized to a normal LD cycle were introduced into DD conditions. At CT 21, the mice were illuminated at 20 lx for 30 min. One hour after illumination, the brains were removed, and 14-μm thick coronal brain sections, including the SCN, were adhered to noncoated glass slides, fixed by treatment with 75% ethanol for 30 s at −18 °C, and then dehydrated. The PixCell IIe Laser Capture Microdissection System (Arcturus) was used to isolate 30 SCN pieces (Supplementary Fig. 1a) from the 4 mouse groups: *PACAP*^*−/−*^ and wild-type mice that were either light stimulated in the late subjective night (CT 21) or kept without light (*n* = 3 per group).

Total RNA was subsequently extracted using the RNeasy MinElute Cleanup Kit (Qiagen) according to the manufacturer’s protocols. RNA amplification and oligonucleotide microarrays were performed as described previously^48,49^, but with several modifications. For the first-round RNA amplification, 15 ng of total SCN RNA pooled from three mouse samples (5 ng per each mouse) was reverse transcribed using Superscript II reverse transcriptase (Invitrogen), and the double-stranded DNA was synthesized with DNA polymerase (Invitrogen). Using the double-stranded DNA as a template, antisense RNA was synthesized using the RiboAmp OA RNA Amplification Kit (Arcturus) according to the manufacturer’s instructions. The second-round amplification was performed similarly, except that the Enzo High Yield RNA Transcript Labeling Kit (Enzo Diagnostics) was used to prepare biotin-labeled antisense RNA. The resulting antisense RNA was hybridized to the GeneChip Mouse Genome 430A array (Affymetrix) using standard methods. Image files were processed using the Microarray Analysis Suite software (Affymetrix).

Microarray data analysis was performed as described previously^48^, with minor modifications. The robust multiarray analysis algorithm was used for background correction, normalization, and expression level summarization. Genes that showed 1.7-fold or greater absolute changes in signal intensity were extracted and classified using the *k*-means clustering algorithm. Functional enrichment analysis and cellular component analysis of the gene clusters was based on gene ontology pathway annotation terms using Data Mining Tool (Affymetrix) algorithm, with *p* values < 0.05 considered statistically significant. The ToppGene Suite (https://toppgene.cchmc.org/) was also used for gene ontology annotation-based functional classification of the genes in the clusters shown in Supplementary Fig. 1b. The gene ontology annotations were cut off at *p* < 0.05 and false discovery rate < 0.05. The gene annotation was limited for the analysis of biological processes (1000 ≤ *n* ≤ 10,000).

Real-time quantitative PCR was performed using amplified RNA from laser-captured microdissected SCNs, Superscript II reverse transcriptase (Invitrogen), and the DyNAmo SYBR Green qPCR Kit (Finnzymes). Primer sequences are shown in Supplementary Table 2. *Gapdh* was amplified as a control.

### In situ hybridization

Brain sections (20 μm-thick) including the SCN were subjected to in situ hybridization performed as described previously^45,50^. A cDNA fragment of mouse *L-Pgds* (GenBank accession number NM_008963.1; nucleotides 2–471) was used as a template to synthesize ^35^S-CTP-labeled cRNA probes. The sections counterstained with cresyl violet clearly revealed morphologically distinct SCNs as a pair of densely aggregated neural cells. In situ hybridization signals of *L-Pgds* was detected as intense clusters of black grains under bright-field illumination as well as white grains under dark-field illumination. We counted the number of *L-Pgds* signals on individual cell bodies that were merged with Nissl-stained neurons in the SCN using ImageJ software (NIH) (Fig. 2b). To quantitatively determine the *L-Pgds*-expressing neurons in the whole SCN, five coronal SCN sections every four sections per mouse were used for statistical analysis.

### Immunohistochemistry

Immunohistochemistry was performed as described previously^9,10^. After in situ hybridization, the sections were incubated with a mouse anti-NeuN antibody (1:1000; Santa Cruz, #sc-246957) or a rabbit anti-Olig2 antibody (1:100; IBL, #18953) a rabbit anti-c-Fos antibody (1:1000; Santa Cruz, #sc-52) overnight at 4 °C. The Vectastain Elite ABC Kit (Vector Lab) was used for immunostaining according to the manufacturer’s protocols. For double immunostaining, we used a 1:1000 dilution of a goat anti-L-PGDS antibody (Santa Cruz, sc-14825), a 1:2000 dilution of a rabbit anti-VIP antibody or a 1:1000 dilution of a rabbit anti-AVP antibody (both anti-VIP and anti-AVP antibodies were kindly provided by Dr. Buijs). After three washes with 0.2% Triton X-100 in PBS, the sections were incubated for 1 h at room temperature in a secondary antibody solution consisting of anti-goat IgG coupled to Alexa-594 (red, 1:200 dilution; #A-11058, Life Technologies) and anti-rabbit IgG coupled to Alexa-488 (green, 1:1000 dilution; #A-11008, Life Technologies) in 0.2% Triton X-100 in PBS with 3% bovine serum albumin. After three washes, the sections were mounted on glass slides with Fluoromount (Diagnostic BioSystems) and dried before imaging. Fluorescent images were captured using a BIO-REVO BZ-9000 fluorescence microscope (Keyence).

### Behavioral study

Light-induced phase shifts in locomotor activity rhythms were examined as described previously^9,10^. Briefly, mice were transferred to DD conditions after being entrained to a 12L:12D cycle, with monitoring of their locomotor activity by far-infrared apparatus (Bio-Medica). After more than 8 days in DD conditions, animals were exposed to a white light pulse of the indicated intensities at CT 15 or CT 21, and their behavioral rhythms were further recorded. The double-plot actograms were produced and the phase shifts were calculated based on the distance between two regression lines drawn from the onset of activity before and after the light pulse using MATLAB (The MathWorks) and the method of Daan and Pittendrigh^9,51^.

Resynchronization to phase shifts of the LD schedule was examined using the jet lag model as follows. After mice were entrained to a 12L:12D cycle with monitoring of their locomotor activity by running wheels, the LD cycle was advanced or delayed by 8 h, and the locomotor activity was further measured for 9 days. The phase shifts were determined as the onset time of activity before and after the shift of the LD cycle.

### Intracerebroventricular injections

Intracerebroventricular injections were performed as described previously^39^. CD-1 mice were anesthetized and placed in a stereotaxic instrument (Narishige). A G-4 cannula (Eicom) was implanted, −0.4 mm posterior, 1.0 mm lateral, and 2.3 mm ventral from the bregma. After cannula implantation, each mouse was given 1 mg/kg buprenorphine (Sigma-Aldrich) to relieve the pain and housed individually for at least 1 week before performing experiments. Thirty minutes before light stimulation, a DP2/CRTH2-selective antagonist, CAY10471, and a DP1 selective antagonist, BW A868C (Cayman Chemical), were diluted with Ringer’s solution (1:100, Fuso Pharmaceutical Industries) and injected at a volume of 2 and 4.6 μl, respectively, at an infusion rate of 1 μl/min, using a microinjection pump (KD Scientific). We previously examined the effect of CAY10471 on DP2/CRTH2 and found that CAY10471 pretreatment before 30 min is sufficient to block DP2/CRTH2 signaling^39^. Zhao et al. previously demonstrated that BW A868C pretreatment for 20 min was sufficient to block DP1 signaling^52^. The day after behavioral experiments, each mouse was intracerebroventricularly injected with 3 μl of 1% (w/v) Evans blue solution (Sigma-Aldrich), and a coronal section of the brain was prepared. The intracerebroventricular injection was judged to be successful if the third ventricle was stained by Evans blue.

### Statistics and reproducibility

Experimental data were analyzed using one-way or two-way analysis of variance (ANOVA). The Tukey–Kramer post hoc test was also performed after significant main effects for drug, time or luminescence intensity were observed. The criterion for statistical significance was *p* < 0.05. Statistical analyses were performed using Stat View software (version 5.0; SAS Institute). Each experiment was repeated at least three times, and sample sizes and numbers are indicated in detail in each figure legend.

### Reporting summary

Further information on research design is available in the Nature Research Reporting Summary linked to this article.

## Supplementary information

Supplementary Information

Description of additional supplementary files

Supplementary Data 1

Reporting Summary

## Data Availability

Microarray data have been deposited to the DDBJ Genomic Expression Archive (GEA) and are available at the accession number E-GEAD-376 and A-GEOD-8299. Data that support the findings of this study are available from the corresponding authors upon reasonable request. Source data underlying plots shown in figures are provided in Supplementary Data 1.

## Code availability

Details of publicly available software used in the study are given in the methods and the Nature Research Reporting Summary linked to this article.
