# Supplementary material for: Lipocalin-type prostaglandin D synthase regulates light-induced phase advance of the central circadian rhythm in mice
Source: Commun Biol. 2020 Oct 8;3:557. doi: 10.1038/s42003-020-01281-w (PMC7544906; doi:10.1038/s42003-020-01281-w)
Supplement: Supplementary file 1 — Supplementary Information [file 42003_2020_1281_MOESM1_ESM.pdf]

Supplementary Information

Lipocalin-type prostaglandin D synthase regulates light-induced phase advance of the central circadian rhythm in mice

Chihiro Kawaguchi, Norihito Shintani, Atsuko Hayata-Takano, Michiyoshi Hatanaka, Ai Kuromi, Reiko Nakamura, Yui Yamano, Yusuke Shintani, Katsuya Nagai, Soken Tsuchiya, Yukihiko Sugimoto, Atsushi Ichikawa, Yasushi Okuno, Yoshihiro Urade, Hiroyuki Hirai, Kin-ya Nagata, Masataka Nakamura, Shuh Narumiya, Takanobu Nakazawa, Atsushi Kasai, Yukio Ago, Kazuhiro Takuma, Akemichi Baba, Hitoshi Hashimoto

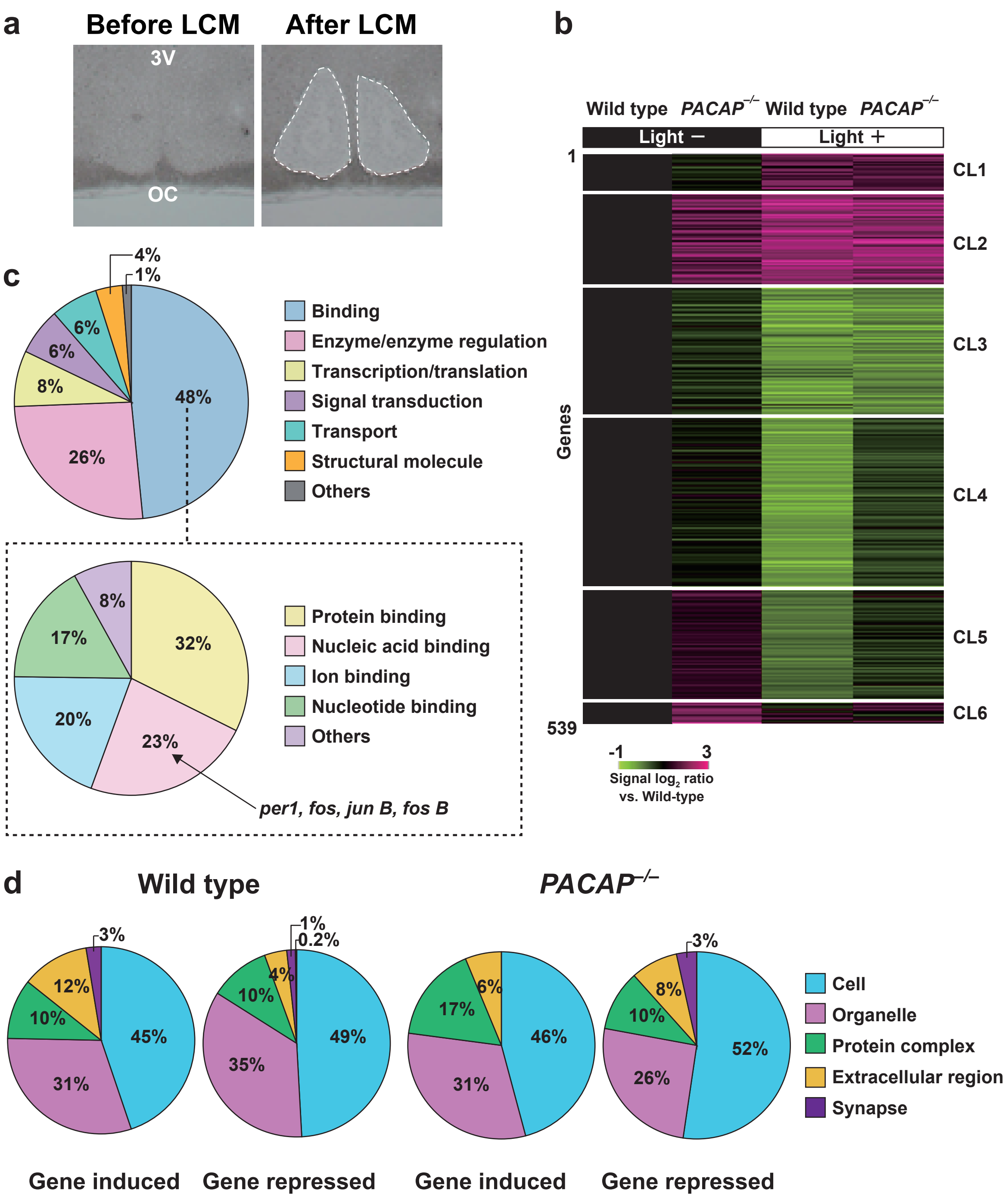

Supplementary Fig. 1. Gene chip analysis of laser capture microdissected SCNs in *PACAP*$^{-/-}$ and wild-type mice illuminated or not illuminated with light in the late subjective night (CT 21).
a, SCN-specific dissection. The photomicrographs were obtained before (left) and after (right) laser capture microdissection. The dotted line shows the dissected areas. 3V, third ventricle; OC, optic chiasma. **b**, mRNA expression levels of the 539 genes with hybridization signal ratios with a more than 1.7-fold change compared to the ratios in wild-type mice kept without light. Colored bars represent the ratio of hybridization measurements as the logarithm (base 2) of fold change vs. wild-type mice without light, according to the scale shown on the bottom left side. Genes were classified into six clusters using the k-means clustering algorithm. **c**, Gene ontology functional classification of light-responsive genes in wild-type mice. **d**, Gene ontology enrichment analysis for cellular components of light-responsive genes in *PACAP*$^{-/-}$ and wild-type mice.

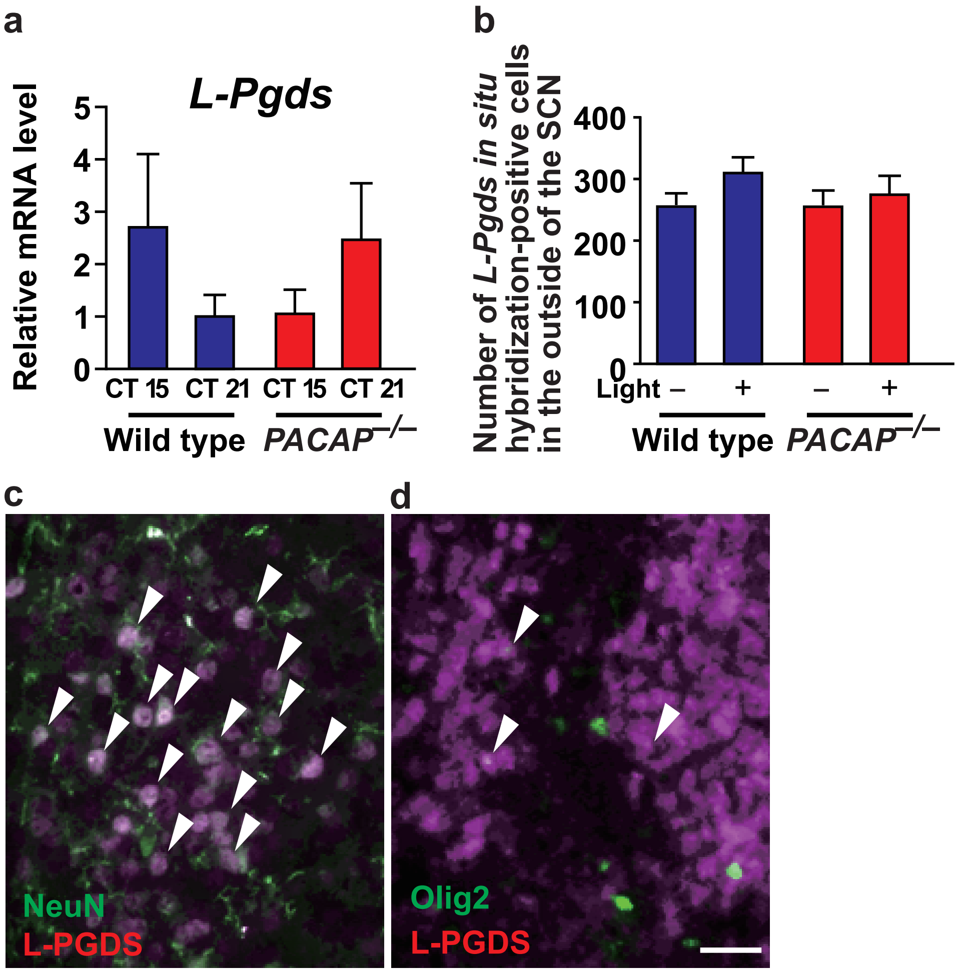

Supplementary Fig. 2. L-PGDS expression in the SCN. a, Real-time quantitative PCR analysis of *L-Pgds* in the SCN upon light stimulation at CT 15 and CT 21 (based on the same data in Fig. 1c, d). The values are shown as the mean ± SEM (n = 4-6 per group). **b,** The number of *L-Pgds* in situ hybridization-positive cells in the outside vicinity of the SCN in the fields of view (650 × 650 μm). Bars, 100 μm.The values are expressed as the mean ± SEM (n = 4 per group). **c** and **d**, Identification of cell types expressing L-PGDS in the SCN. White arrowheads indicate the cells expressing L-PGDS (magenta) and NeuN (green, **c**) or Olig2 (green, **d**). Bar, 50 μm.

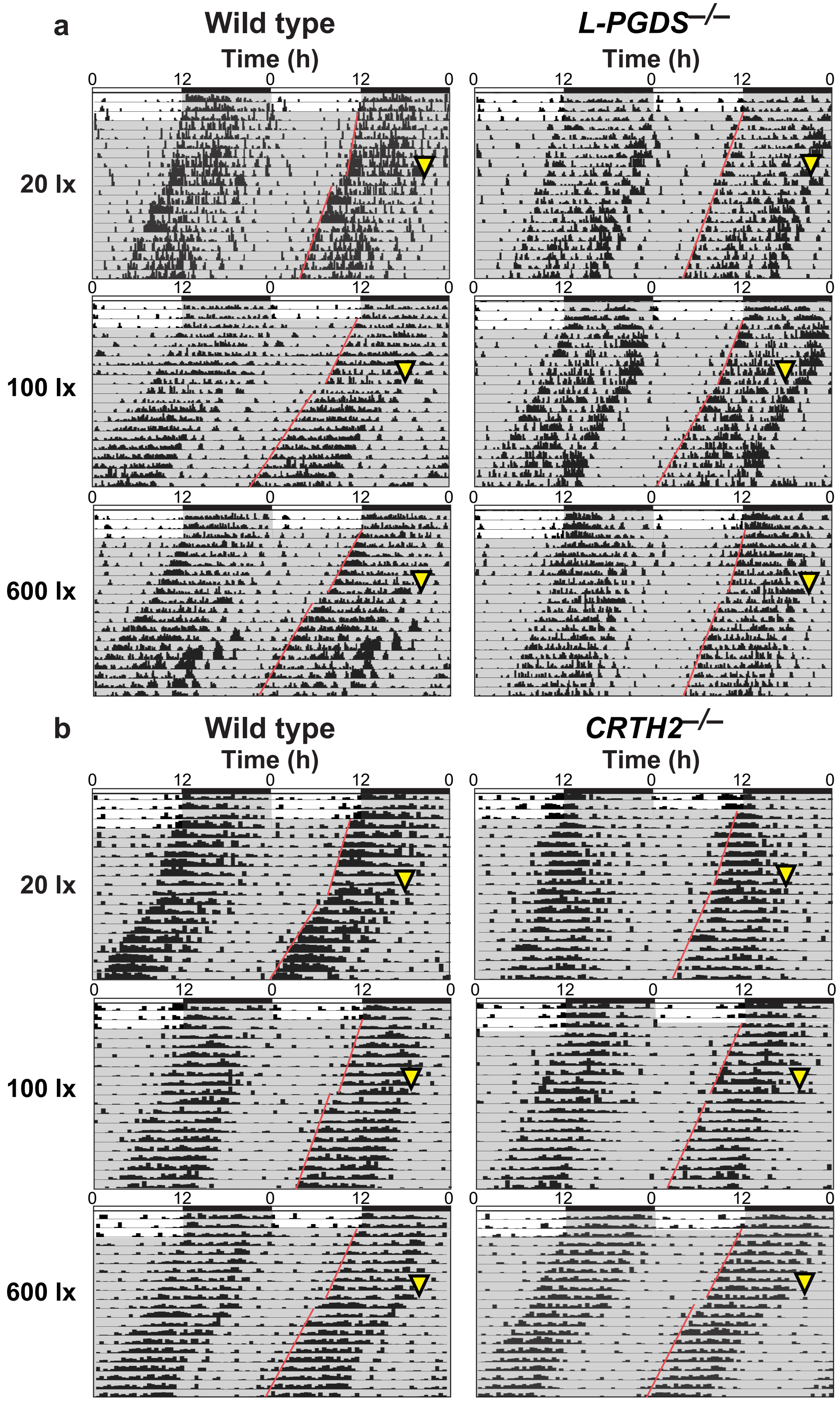

Supplementary Fig. 3. Impairment in light-induced phase advance in *L-PGDS*$^{-/-}$ and *CRTH2*$^{-/-}$ mice. Representative double-plotted actograms in *L-PGDS*$^{-/-}$ (**a**) and *CRTH2*$^{-/-}$ (**b**) mice are shown. Mice were illuminated with light (20, 100, 600 lx) at CT 21 (yellow arrowheads). Paired red lines represent the onset of activity.

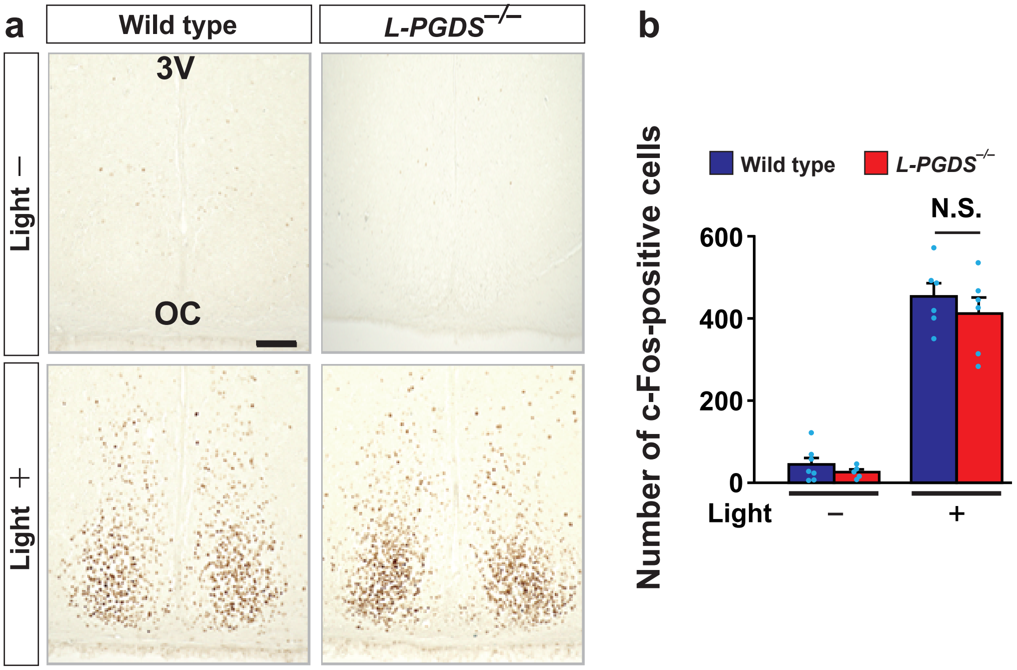

Supplementary Fig. 4. c-Fos expression induced by light stimulation in *PACAP*$^{-/-}$ and wild-type mice. a, Representative photomicrographs of c-Fos immunoreactivity in the SCN 1 hour after light stimulation (30 min, 20 lx) at CT 21. Bar, 100 μm. 3V, third ventricle; OC, optic chiasma. **b,** The number of c-Fos-immunoreactive cells in the SCN was counted. The values are shown as the mean ± SEM (n = 5-7 per group). Statistically significant differences were assessed using two-way ANOVA.

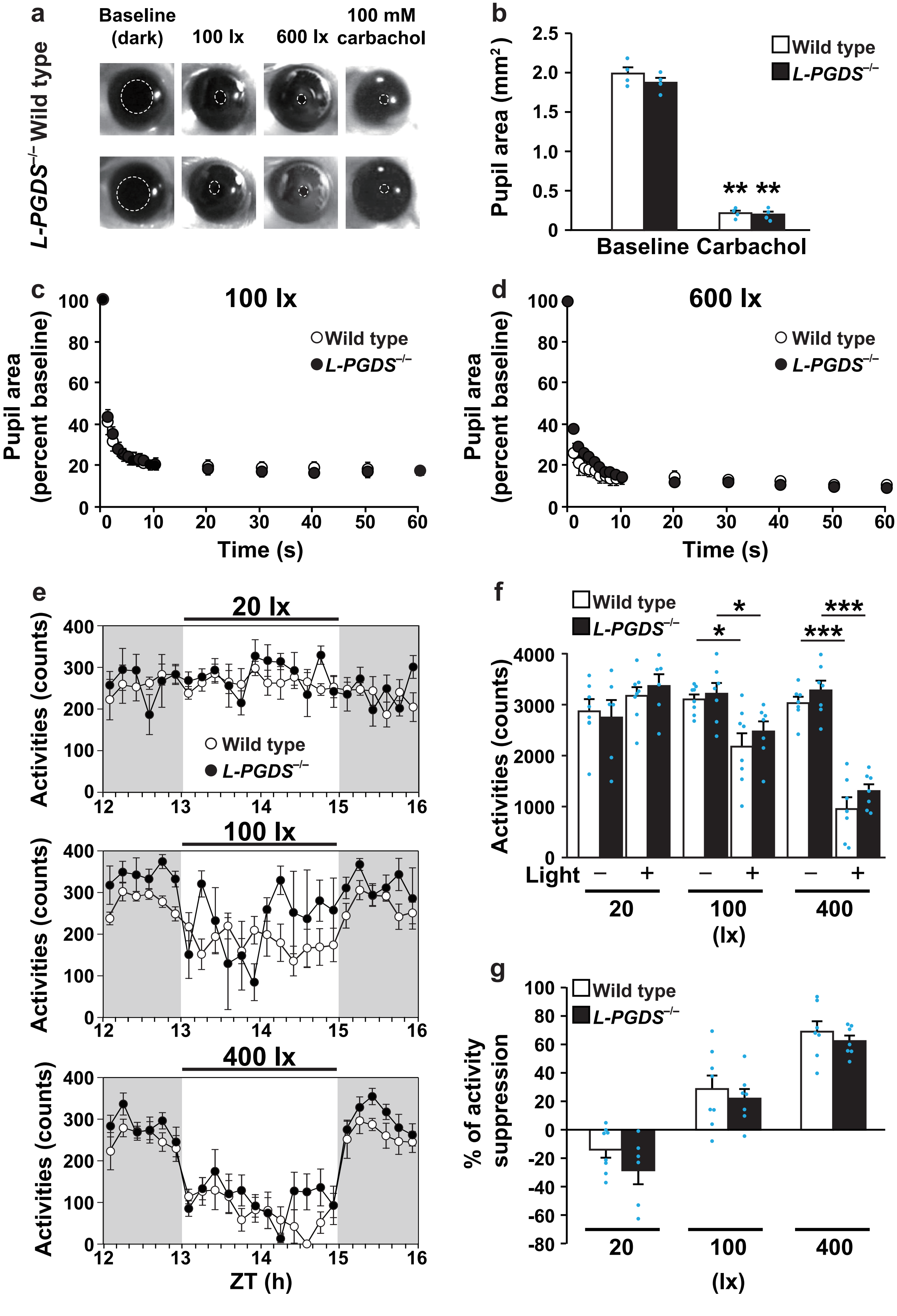

Supplementary Fig. 5 *(legend on next page)*

Supplementary Fig. 5. *L-PGDS*$^{-/-}$ mice show a normal pupillary light reflex and negative masking responses to light. a-d, Pupillary light reflex. **a,** Representative photographs of pupillary constriction 1 min post-irradiation. Baseline (dark), scotopic conditions; carbachol, 1 min after topical instillation of carbachol under dark conditions. White dashed lines indicate pupillary diameters. **b,** Pupil sizes at baseline and 1 min after topical instillation of carbachol. **c** and **d**, Time courses of pupillary miosis for 1 min after irradiation with light of 100 lx (**c**) and 600 lx (**d**). The graphs indicate normalized pupil area relative to time 0. **e–g**, Negative masking responses to light. **e**, Wheel-running activities for ZT 12-16 in mice that were illuminated with light (20, 100 or 400 lx) during ZT 13-15. **f**. Total activities during dark and light illumination. **g**, Light-induced locomotor suppression determined as the ratios of total activity in the dark vs. those under light stimulation. The values are shown as the mean ± SEM (n = 4 (**b–d**), n = 3-8 (**e–g**) per group). *$p < 0.05$, **$p < 0.01$, ***$p < 0.001$. Statistically significant differences were assessed using two-way ANOVA and Tukey-Kramer tests.

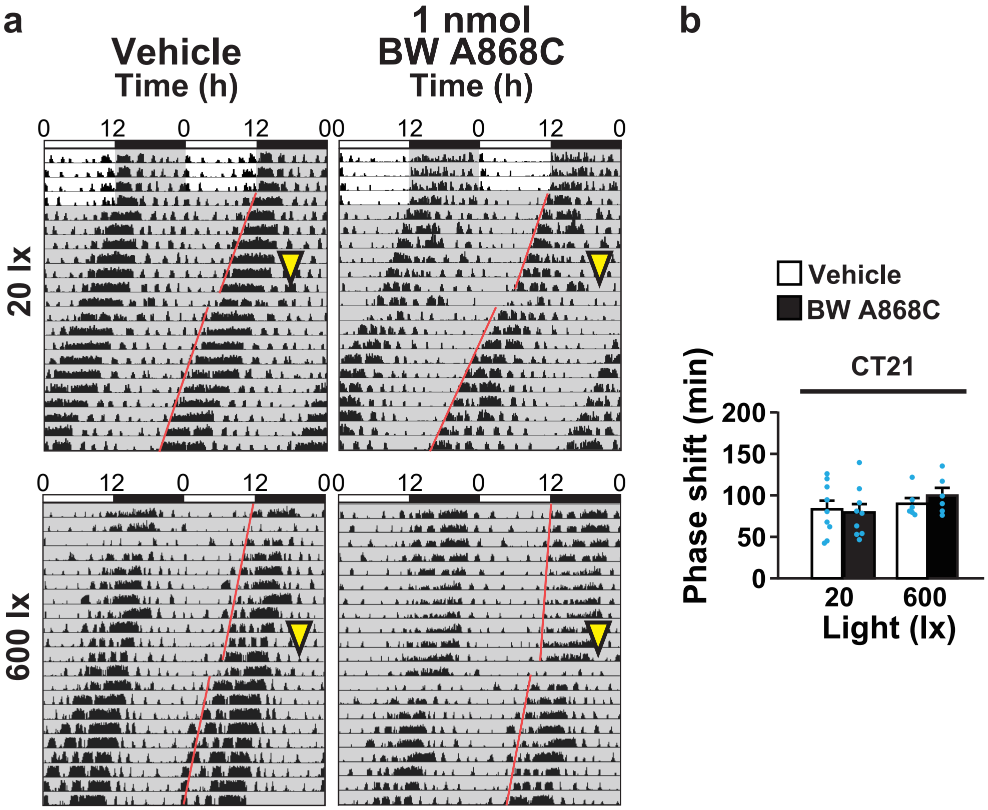

Supplementary Fig. 6. Light-induced phase advance in mice administered the DP1 blocker BW A868C. Phase shift induced by light stimulation at CT 21 was examined in CD-1 wild-type mice administered the DP1 blocker BW A868C or a vehicle (Ringer' s solution) 30 min before light stimulation. **a,** Representative double-plotted actograms. **b,** Quantification of the phase shift. The values are expressed as the mean ± SEM (n = 6-9 per group). Statistically significant differences were assessed using two-way ANOVA. Yellow arrowheads indicate light stimulation (20 lx or 600 lx, 30 min). Paired red lines represent the onset of activity.

Supplementary Table 1. Functional gene ontology analysis of the genes in the clusters. Gene ontology (GO) annotations (molecular function) for each cluster (Supplementary Fig. 1b) were obtained using the ToppGene Suite[1] (https://toppgene.cchmc.org/) at cutoffs of $p < 0.05$ and FDR < 0.05. The genes in the annotation were limited for the analysis of biological process (1,000 ≤ n ≤ 10,000), and the top five GO terms are shown.

Cluster	ID	Name	*p*-value	FDR q value	Number of genes
**1**	GO:0044212	Transcription regulatory region DNA binding	2.23E-5	2.69E-4	10
	GO:0001067	Regulatory region nucleic acid binding	2.33E-5	2.69E-4	10
	GO:0003690	Double-stranded DNA binding	2.57E-5	2.69E-4	10
	GO:0043565	Sequence-specific DNA binding	8.06E-5	6.89E-4	10
	GO:0140110	Transcription regulator activity	9.74E-5	7.49E-4	15
**2**	GO:0019899	Enzyme binding	4.56E-6	2.82E-4	27
	GO:0042802	Identical protein binding	8.95E-6	2.82E-4	22
	GO:0008092	Cytoskeletal protein binding	1.11E-3	1.23E-2	12
	GO:0140096	Catalytic activity, acting on a protein	1.17E-3	1.23E-2	26
	GO:0005102	Signaling receptor binding	2.27E-3	1.89E-2	16
**3**	GO:0003723	RNA binding	2.40E-6	6.64E-4	30
	GO:0019899	Enzyme binding	2.06E-3	4.74E-2	29
**4**	GO:0097159	Organic cyclic compound binding	7.16E-6	1.68E-4	84
	GO:1901363	Heterocyclic compound binding	8.02E-6	1.68E-4	83
	GO:0003723	RNA binding	2.32E-5	3.66E-4	35
	GO:0003676	Nucleic acid binding	4.83E-4	5.39E-3	58
	GO:0017111	Nucleoside-triphosphatase activity	5.14E-4	5.39E-3	25
**5**	GO:0003723	RNA binding	6.37E-5	1.49E-2	25

Supplementary Table 2. Primers used for RT-PCR.

Gene	Primer sequence (5’ to 3’)	Target fragment size (bp)	Annealing temperature (℃)
Per1	agcgcatccactctggtta<br>aggcagcttggtgtgtgtc	81	60
Prok2	cggaggatgcaccacacc<br>ccggttgaaagaagtccttaaaca	73	58
Ptgds	tcaacaagacaagttcctgg<br>tgaatttctccttgagctcg	390	60
Cryab	ttcttcggagagcacctgtt<br>ccccagaaccttgactttga	207	57
Gapdh	gtgttccctaccccaatgtg<br>taccaggaaatgagcttgac	242	60

Supplementary methods

Masking responses to light

Masking responses to light were examined as previously described[2,3]. Briefly, mice were entrained to a LD cycle with monitoring of their locomotor activity by running wheels. A light pulse was given to the animals for 2 h from ZT 13. The percentage of activity suppression during the first hour after the light stimulation was calculated using the following formula[4]: % activity suppression = (B – A)/B × 100, where A is the amount of activity during the first hour after ZT 13 on the day on which light stimulations was given, and B is the average activity level during a comparable time period during the previous three days.

Pupillary response

After the mice were dark adapted for at least 1 h, they were exposed to 1 min of white light (light intensities: 100 or 600 lx) without anesthesia. Temporal changes in pupillary responses were archived through an infrared video recorder. All tests were conducted between ZT 3 and ZT 7. Pupil constriction was quantified as described previously[5]. To confirm the intrinsic function of the pupillary sphincter, the effect of parasympathetic activation by topical administration of carbachol (1 M, Sigma-Aldrich) on pupil constriction was assessed.

Supplementary references

1 Chen J., Xu H., Aronow B.J. & Jegga A.G. Improved human disease candidate gene prioritization using mouse phenotype. *BMC Bionformatics.* **8**, 392 (2007).

2 Kawaguchi, C. *et al.* Changes in light-induced phase shift of circadian rhythm in mice lacking PACAP. *Biochem. Biophys. Res. Commun.* **310**, 169-175 (2003).

3 Kawaguchi, C. *et al.* PACAP-deficient mice exhibit light parameter-dependent abnormalities on nonvisual photoreception and early activity onset. *PLoS One* **5**, e9286 (2010).

4 Panda, S. *et al.* Melanopsin is required for non-image-forming photic responses in blind mice. *Science* **301**, 525-527 (2003).

5 Lucas, R. J., Douglas, R. H. & Foster, R. G. Characterization of an ocular photopigment capable of driving pupillary constriction in mice. *Nat. Neurosci.* **4**, 621-626 (2001).
