# Supplementary material for: Lipocalin-type prostaglandin D synthase regulates light-induced phase advance of the central circadian rhythm in mice
Source: Commun Biol. 2020 Oct 8;3:557. doi: 10.1038/s42003-020-01281-w (PMC7544906; doi:10.1038/s42003-020-01281-w)
Supplement: Supplementary file 4 — Reporting Summary [file 42003_2020_1281_MOESM4_ESM.pdf]

nature research

Corresponding author(s): Hitoshi Hashimoto and Atsuko Hayata-Takano

Last updated by author(s): 2020/07/17

Reporting Summary

Nature Research wishes to improve the reproducibility of the work that we publish. This form provides structure for consistency and transparency in reporting. For further information on Nature Research policies, see our Editorial Policies and the Editorial Policy Checklist.

Statistics

For all statistical analyses, confirm that the following items are present in the figure legend, table legend, main text, or Methods section.

n/a	Confirmed	
☐	☒	The exact sample size (n) for each experimental group/condition, given as a discrete number and unit of measurement
☐	☒	A statement on whether measurements were taken from distinct samples or whether the same sample was measured repeatedly
☐	☒	The statistical test(s) used AND whether they are one- or two-sided<br>*Only common tests should be described solely by name; describe more complex techniques in the Methods section.*
☒	☐	A description of all covariates tested
☐	☒	A description of any assumptions or corrections, such as tests of normality and adjustment for multiple comparisons
☐	☒	A full description of the statistical parameters including central tendency (e.g. means) or other basic estimates (e.g. regression coefficient) AND variation (e.g. standard deviation) or associated estimates of uncertainty (e.g. confidence intervals)
☐	☒	For null hypothesis testing, the test statistic (e.g. F, t, r) with confidence intervals, effect sizes, degrees of freedom and P value noted<br>*Give P values as exact values whenever suitable.*
☒	☐	For Bayesian analysis, information on the choice of priors and Markov chain Monte Carlo settings
☒	☐	For hierarchical and complex designs, identification of the appropriate level for tests and full reporting of outcomes
☒	☐	Estimates of effect sizes (e.g. Cohen's d, Pearson's r), indicating how they were calculated

Our web collection on statistics for biologists contains articles on many of the points above.

Software and code

Policy information about availability of computer code

Data collection	PixCell IIe Laser Capture Microdissection System (biochemistry), CFX96 real-time PCR detection system (biochemistry), BIO-REVO BZ-9000 (biochemistry), far-infrared apparatus (behavior) were used for data collection.
Data analysis	Microarray Analysis Suite software, ToppGene Suite (https://toppgene.cchmc.org/), Stat-View (version 5.0), NIH ImageJ (version 1.5b), MATLAB and Photoshop Elements 9 were used to analyze the data.

For manuscripts utilizing custom algorithms or software that are central to the research but not yet described in published literature, software must be made available to editors and reviewers. We strongly encourage code deposition in a community repository (e.g. GitHub). See the Nature Research guidelines for submitting code & software for further information.

Data

Policy information about availability of data

All manuscripts must include a data availability statement. This statement should provide the following information, where applicable:

- Accession codes, unique identifiers, or web links for publicly available datasets
- A list of figures that have associated raw data
- A description of any restrictions on data availability

Microarray data have been deposited to the DDBJ Genomic Expression Archive (GEA) and are available at the accession number E-GEAD-376 and A-GEOD-8299. The datasets generated during the current study are available from the corresponding authors on reasonable request.

Field-specific reporting

Please select the one below that is the best fit for your research. If you are not sure, read the appropriate sections before making your selection.

☒ Life sciences ☐ Behavioural & social sciences ☐ Ecological, evolutionary & environmental sciences

For a reference copy of the document with all sections, see nature.com/documents/nr-reporting-summary-flat.pdf

Life sciences study design

All studies must disclose on these points even when the disclosure is negative.

Sample size	We did not use statistical methods to predetermine the sample sizes, but the sample sizes in this study are similar to those generally employed in this field.
Data exclusions	All obtained data were included in the analysis.
Replication	All studies have been repeated with similar results. We included detailed methods in the manuscript.
Randomization	No randomization was employed in this study.
Blinding	All histological, cell biological, behavioral and electrophysiological experiments were blindly performed.

Reporting for specific materials, systems and methods

We require information from authors about some types of materials, experimental systems and methods used in many studies. Here, indicate whether each material, system or method listed is relevant to your study. If you are not sure if a list item applies to your research, read the appropriate section before selecting a response.

Materials & experimental systems

n/a	Involved in the study	
☐	☒	Antibodies
☒	☐	Eukaryotic cell lines
☒	☐	Palaeontology and archaeology
☐	☒	Animals and other organisms
☒	☐	Human research participants
☒	☐	Clinical data
☒	☐	Dual use research of concern

Methods

n/a	Involved in the study	
☒	☐	ChIP-seq
☒	☐	Flow cytometry
☒	☐	MRI-based neuroimaging

Antibodies

Antibodies used

Mouse anti-NeuN (Santa Cruz, #sc-246957, 1:1000)
Rabbit anti-Olig2 (IBL, #18953, 1:100)
Rabbit anti-c-Fos (Santa Cruz, #sc-52, 1:1000)
Goat anti-L-PGDS (Santa Cruz, sc-14825, 1:1000)
Rabbit anti-AVP (was kindly provided by Dr. Buijs from Nether lands Institute for Brain Research, 1:1000)
Rabbit anti-VIP (was kindly provided by Dr. Buijs from Nether lands Institute for Brain Research, 1:2000)
Biotinylated goat anti-rabbit IgG (Vector Labs, CA, USA, #BA-1000, 1:200)
Biotinylated goat anti-mouse IgG (Vector Labs, #BA-9200, 1:200)
Biotinylated horse anti-goat IgG (Vector Labs, #BA-9500, 1:200)
Alexa Fluor 488-conjugated goat anti-rabbit IgG (Life Technologies, CA, USA, #A-11008, 1:200)
Alexa Fluor 594-conjugated donky anti-goat IgG (Life Technologies, CA, USA, #A-11058, 1:200)

Validation

The antibodies were purchased by qualified vendors that provided a validation on the manufacturer's website.
Anti-AVP and VIP were kindly provided by Dr. Buijs from Nether lands Institute for Brain Research.
The validation of these two antibody was provided the reference manuscript (Buijs RM., et al. 1989, Biomed. Res., 10(3);213-221).

Animals and other organisms

Policy information about studies involving animals; ARRIVE guidelines recommended for reporting animal research

Laboratory animals

All experiment was carried out on male mice at 2-4 months of age. The generation of PACAP, L-PGDS, DP1, and CRTH2 deficient mice were back-crossed for at least 10 generations onto the CD-1 (PACAP), C57BL/6 (DP1) or BALB/c (L-PGDS and CRTH2) genetic background.

Wild animals	The study did not involve wild animals.
Field-collected samples	The study did not involve samples collected from field.
Ethics oversight	All animal care and handling procedures were approved by the Animal Care and Use Committee of the Graduate School of Pharmaceutical Sciences, Osaka University.

Note that full information on the approval of the study protocol must also be provided in the manuscript.
